# Measurement of Racial Microaggressions During Pregnancy and While Receiving Prenatal Care: a Validation Study of the Daily Life Experiences Scale Among Black Postpartum Women

**DOI:** 10.1007/s40615-025-02777-z

**Published:** 2025-12-08

**Authors:** Sarah C. Haight, Dawn P. Misra, Lisa Miller, Mercedes Price, Jaime C. Slaughter-Acey

**Affiliations:** 1 Department of Epidemiology and Biostatistics, College of Public Health, University of Georgia, 101 Buck Road, Athens, GA 30606, USA; 2 Department of Epidemiology and Biostatistics, Michigan State University, 909 Wilson Road, East Lansing, MI 48824, USA; 3 Department of Epidemiology, Gillings School of Global Public Health, University of North Carolina, 135 Dauer Drive, Chapel Hill, NC 27599, USA; 4 Carolina Population Center, University of North Carolina, 123 W Franklin Street, Chapel Hill, NC 27516, USA

**Keywords:** Discrimination, Microaggressions, Perinatal, Postpartum, Psychometric, Racism

## Abstract

**Objective:**

The Daily Life Experiences scale measures the frequency of experiences of racial microaggressions (everyday actions or comments expressing prejudice). We assessed the factor structure, reliability, and validity of the DLE-F among Black postpartum women in two contexts: (1) daily life (DLE-F) and (2) while seeking or receiving prenatal care (DLE-PNC-F).

**Methods:**

Data were from the LIFE-2 cohort of Black women delivering in Detroit, Michigan from 2023 to 2025. Questionnaires were completed during postpartum hospitalization. The 20-item DLE-F assessed past 3-month experiences of microaggressions; the adapted 17-item DLE-PNC-F referenced experiences in prenatal care. Confirmatory factor analysis (CFA) was used to evaluate structure. Internal consistency was assessed with Cronbach’s alpha. Convergent validity was tested via correlations with major discrimination, racism-related stress, vigilance, depressive symptoms, and perceived stress. Discriminant validity was tested against self-esteem, self-kindness, and collective hope.

**Results:**

Among 514 postpartum women, both the DLE-F and DLE-PNC-F followed a unidimensional structure with modest fit and strong internal consistency (α = 0.97). The scales were moderately correlated (ρ = 0.51), suggesting they capture related, but distinct domains. The DLE-F showed convergent validity with major discrimination (ρ = 0.53) and racism-related stress (ρ = 0.46). Neither scale was convergent with depressive symptoms or perceived stress. Both scales demonstrated low correlations with self-esteem, self-kindness, and collective hope, supporting discriminant validity.

**Conclusions:**

We validate the original DLE-F and a newly developed healthcare-specific version, the DLE-PNC-F among postpartum Black women. Results from this study provide evidence for the use of the DLE among Black postpartum women, while also highlighting the need to further develop a modified version with items specifically relevant to experiences during prenatal care.

## Introduction

Racism is a fundamental determinant of health that operates across multiple levels: structural, institutional, and interpersonal [1, 2]. Yet, our ability to document the health impacts of racism depends upon having reliable, valid tools to measure how racial discrimination is experienced. Experiences of racial discrimination can range from overt acts of racial discrimination (e.g. racial slurs) [3] to more subtle acts such as jokes. Microaggressions are defined as brief, but commonplace verbal or behavioral insults directed towards racially minoritized individuals [4]. Though often subtle or ambiguous, microaggressions can accumulate over time to affect health through increased stress, strained clinical interactions, and reduced quality of care [5–7]. Despite a growing interest in capturing this subtle form of discrimination in both research and clinical contexts, existing measures of racial microaggressions are rarely validated outside of young adult or college student populations, and often overlook the specific ways that microaggressions are experienced in healthcare encounters. It is important to understand how experiences of discrimination are operationalized and whether instruments that measure them perform consistently across population and settings. The use of psychometrically validated tools is critical to accurately assess the impacts of racism-related experiences on health and well-being.

The Daily Life Experiences (DLE) scale, developed by Shelly Harrell (1997) as part of the Racism and Life Experiences Scales (RaLES), was designed to assess the frequency of racial microaggressions as a form of racism-related stress [8]. The DLE has been used in numerous studies to investigate the effects of daily racial discrimination on health outcomes [9–15]. Early validation work, unpublished by Harrell (1997), supported a unidimensional factor structure for the DLE and psychometric reliability and validity [8]. More recently, Lee and colleagues (2021) revisited the factor structure and validity among Black college students, finding support for a unidimensional structure aligning with Harrell’s work, despite some indication of two sub-factors: microinvalidations and overt discrimination [8, 16]. The DLE demonstrated good internal consistency and convergent validity, correlating with measures of everyday discrimination, depressive symptoms, anxiety symptoms, and racial identity (measured by the Multidimensional Inventory of Black Identity [17]). Importantly, Lee et al. also established measurement invariance across gender and called for validation across a broader range of life stages and social context, particularly outside the college environment, where the form, frequency, and impact of racial microaggressions may differ [16]. To date, no additional studies have validated the scale, meaning that it remains largely unvalidated for populations other than college students.

To address this gap in the literature, we utilize post-delivery survey data to understand how the scale functions among Black postpartum women living in the United States. Numerous studies have shown that for Black women, experiences of racial discrimination, especially in the form of subtle, everyday microaggressions, are linked to chronic stress and poorer mental and physical health outcomes [18]. These experiences are especially consequential during the perinatal period, a time marked by increased physiological vulnerability and intensive engagement with healthcare services [19, 20]. Prior research has shown that pregnant and postpartum individuals, especially Black women, report heightened experiences of discrimination while seeking and receiving perinatal health care [21, 22]. These experiences include both explicit discrimination [23, 24] and microaggressions such as dismissal, stereotyping, or being talked down to [25]. This dynamic can erode trust and communication [26–29], leading to reluctance to ask questions and delayed or declined care [18, 21, 22, 29, 30]. However, most of these studies have relied on either a single-item question or general measures like the DLE, without considering whether these tools adequately capture experiences of microaggressions *specific* to seeking and receiving healthcare. Using a generalized measure without assessing its contextual performance may obscure important differences in how microaggressions experienced during healthcare impact outcomes.

This study addresses the aforementioned gaps in validation samples and settings by using post-delivery survey data among Black women in Detroit, Michigan to (1) assess the factor structure, reliability, and validity of the DLE for Black postpartum women, but also (2) test a modified version of the DLE that specifically asks about microaggressions experienced while seeking and receiving prenatal care. This approach allows us to assess whether the DLE performs consistently across contexts and whether a healthcare-specific adaptation is warranted for perinatal populations. By validating both the DLE for use in the post-delivery period as well as a prenatal-care specific DLE, we aim to support future research examining the effects of racism-related stress on perinatal health and wellbeing.

## Methods

### Data and Sample

Data were from the Life Course Influences on Fetal Environments (LIFE-2) study. LIFE-2 is an ongoing cohort study of Black women (ages 18 to 45 years) who recently (2023–2025) gave birth to a single live-born infant in Metropolitan Detroit, Michigan. Inclusion criteria include self-identifying as Black or African American, being born in Michigan after 1987, and delivering at one of two participating birthing hospitals in Metropolitan Detroit, Michigan. Women are recruited in the immediate post-delivery period (24 to 48 h) in the postpartum hospital unit. Among all respondents up to July 2025 (n = 592), the analytical sample included all respondents with complete data on either of the DLE scales (n = 514).

### Measures

The Daily Life Experiences frequency scale (DLE-F) is a 20-item scale that measures the frequency of racial microaggressions experienced across general daily life, without reference to any specific setting or domain (e.g. being ignored, overlooked or not given service; being stared at by strangers). Participants are asked “tell us how often you have experienced each of the following situations as a result of your race, skin color, or racism, during the past 3 months,” with response options ranging from ‘never’ to ‘once a week or more’.

We evaluated two versions of the DLE-F. First, the full 20-item DLE-F which captures experiences across broad, nonspecific daily context. Second, we modified the scale to specifically assess experiences while seeking and receiving prenatal care (DLE-PNC-F), asking respondents “please tell us if any of these things ever happened to you while seeking or receiving prenatal care,” with response options ranging from ‘never’ to ‘once a week or more during my pregnancy.’ In the DLE-PNC-F, 17 of the original 20 items were retained; three items (items 10, 18, and 20) were removed because they were not applicable to the clinical setting (e.g., others expecting your work to be inferior). Both the DLE-F and the DLE-PNC-F were maintained as continuous and ranged from 20–120 and 17–102, respectively.

### Analysis

Factor structure was determined via confirmatory factor analysis (CFA). CFA was used because the DLE has been shown to follow a unidimensional structure [8, 16] and we sought to confirm this structure in the immediate postpartum period. We used a weighted least square mean and variance-adjusted estimator (WLSMV) since the data were ordinal [31, 32]. Related, all presented estimates were standardized or robust. Model fit was determined to be good if the root mean square error of approximation (RMSEA) was ≤ 0.07, comparative fit index (CFI) >0.90, Tucker-Lewis Index (TLI) >0.90, and standardized root mean square residual (SRMR) <0.06 [31, 32]. If model fit was deemed poor, the factor structure was re-evaluated to determine whether a unidimensional structure was most appropriate. The main analysis used the entire sample, but we performed a split-sample cross-validation to confirm that the measurement was not overfit to the data. We randomly assigned observations to two subsamples: development and validation and chi-square and two-sample t-tests were used to determine balance of characteristics. The development sample was used to estimate the CFA and the model was replicated in the validation sample. Model fit of the development and validation samples were compared to confirm the robustness of the measurement.

The correlation between the DLE-F and DLE-PNC-F was calculated with Spearman’s correlation coefficient. We also calculated the correlation between the DLE-PNC-F and a version of the DLE-F with the same 17 items as the DLE-PNC-F. Spearman’s correlation coefficients were used because of the ordinal nature of the responses that make up these scales. Internal consistency for both scales was determined via Cronbach’s Alpha.

Convergent validity, or the extent to which the measure correlates with a previously validated measure designed to assess a similar or related construct [33], was assessed by comparing the DLE scales to similar measures (major experiences of discrimination) and related constructs (vigilance, anticipatory racism-related stress, stress, and depressive symptoms). The latter were chosen in response to theoretical models of discrimination-stress [34] and racism-related stress [35] which posit that discrimination can cause increased vigilance, both of which can be a significant source of a stress, which over time harms mental health [36]. Discriminatory validity, or the extent to which a measure is unique to potentially related, but distinct concepts was assessed by comparing the DLE scales to scales measuring self-esteem, self-kindness, and collective hope. These constructs were chosen to be proxies for factors like optimism and personality which are thought to influence survey response habits. If divergent validity is demonstrated, that indicates that the DLE successfully measures a concept like microaggressions beyond factors that may influence how an individual responds to surveys, like positivity. Specific details on the comparison scales can be found in Online Resource 1. The absolute value of the correlation between the measures was calculated using Spearman’s correlation coefficients. To determine whether values reflected convergent and discriminant validity, we referred to Nunnally (1987) who states that high convergence will be represented by “highly correlated” measures, whereas measures that are correlated “near zero” suggest weak or no convergence. [37] As ‘high’ is typically undefined, we used cut-offs from the field of psychology and considered strong or moderate correlations (rho ≥ 0.4) to be evidence of strong or moderate convergent validity, respectively, and zero or weak correlations (rho<0.4) to be evidence of strong or moderate discriminant validity, respectively [38]. Statistical significance was defined as p < 0.05. All analyses were conducted in R (Version 9.1.401).

## Results

Among this sample of 514 postpartum women, most were 26–30 years of age (36.0%), unmarried (57.2%), received at least a high school diploma (83.5%), reported a family income of <$10,000 (32.7%), and sought prenatal care (93.2%; Table 1). Average scores on the DLE scales were 39.1 for the DLE-F (SD = 20.7) and 24.6 for the DLE-PNC-F (SD = 14.9). The DLE-F and the DLE-PNC-F were moderately correlated (rho = 0.51), and this held true when the DLE-F was restricted to the same 17 items as the DLE-PNC-F (rho = 0.51).

### Daily Life Experiences (DLE)

The one-factor CFA for the DLE-F indicated that some items (5, 6, and 7) could benefit from allowing correlations between them. However, there was insufficient evidence to depart from a unidimensional structure. After accounting for these correlations, the one-factor model demonstrated modest fit (*X*^2^=889.72; RMSEA = 0.13; CFI/TLI = 0.90/0.88; SRMR = 0.04; Table 2), strong internal consistency (α=0.97), and all standardized factor loadings were > 0.70 (Table 3). The scale displayed moderate convergent validity with major experiences of discrimination (rho = 0.53) and anticipatory racism-related stress (rho = 0.46), but not with vigilance (rho = 0.36) or the mental health scales for depression (rho = 0.21) or stress (rho = 0.24; Table 4). The DLE displayed strong discriminant validity with scales measuring self-esteem (rho = 0.14), self-kindness (rho = 0.17), and collective hope (rho=0.07).

### Daily Life Experiences in Reference to Prenatal Care (DLE-PNC-F)

The one-factor CFA for the DLE-PNC-F (experiences while seeking and receiving prenatal care) demonstrated modest fit (*X*^2^=568.87; RMSEA = 0.19; CFI/TLI = 0.88/0.86; SMSR = 0.03; Table 2), strong internal consistency (α=0.97), and all standardized factor loadings were > 0.80 (Table 3). The scale did not display convergent validity with the major experiences of discrimination scale (rho = 0.38), vigilance (rho = 0.30), anticipatory racism-related stress (rho = 0.28), or the mental health scales for depression (rho = 0.23) or stress (rho = 0.17; Table 4). The DLE-PNC-F displayed strong discriminant validity with self-esteem (rho = 0.15), self-kindness (rho = 0.15), and collective hope (rho=0.06).

### Cross-Validation

The cross-validation subsamples were balanced in terms of age, marital status, education, income, DLE scores, and all comparison scales (all p-values > 0.05). The development subsample mirrored the results found in the overall study and findings remained robust in the validation subsample. For the DLE-F, the one-factor model including correlations for items 5, 6, and 7 had modest fit (*X*^2^=508.41; RMSEA = 0.16; CFI/TLI = 0.86/0.84; SMSR = 0.04), strong internal consistency (α =0.97), and all standardized factor loadings were > 0.80 (data not shown). For the DLE-PNC-F, the one-factor model had modest fit (*X*^2^=299.94; RMSEA=0.23; CFI/TLI=0.85/0.83; SMSR=0.03), strong internal consistency (α =0.97), and all standardized factor loadings were > 0.80.

## Discussion

This study assessed the factor structure, reliability, and validity of two versions of the Daily Life Experiences scale among Black women during their postpartum hospitalization. We assessed both the original DLE-F, which measures general microaggressions across nonspecific daily contexts, and a modified version adapted to experiences specifically encountered while seeking or receiving prenatal care (DLE-PNC-F). Across both settings, the DLE scales demonstrated a single-factor structure with modest fit, strong internal consistency, moderate convergent validity, and strong discriminant validity in our sample of Black mothers in the days following the birth of their child. Results from this study provide evidence for the use of the DLE among Black postpartum women, while also highlighting the need to further develop a modified version with items specifically relevant to experiences during prenatal care.

Our finding that both measures followed a unidimensional structure is consistent with the formative work by Harrell [8] and a recent validation study among Black college students [16]. Although Lee et al. observed some evidence for a bifactor model distinguishing invalidation and overt discrimination, they ultimately concluded that the single-factor model was appropriate. The emergence of a unidimensional structure in our study, even when focusing specifically on the healthcare setting, suggests that the DLE items primarily capture a general experience of race-related microaggressions. Theoretical frameworks, such as Sue et al.’s categorization of microaggressions into three types: microinsults (rude or insensitive remarks), microassaults (explicit and often verbal attacks), and microinvalidations (comments or behaviors that dismiss or negate a person’s lived experience), suggest that microaggressions may be multidimensional [39]. It is possible that the existing items in the DLE are not sufficiently nuanced to differentiate between these subtypes of microaggressions, contributing to the consistent finding of a single factor across populations and contexts. Future work should consider expanding the DLE to incorporate additional items that differentiate between subdimensions such as invalidation and overt discrimination, as this would offer a more granular understanding of how different types of microaggressions impact health and well-being.

The DLE-F demonstrated convergent validity via significant correlations with measures of experiences of discrimination and anticipatory race-related stress, aligning with prior validation work [16]. Our findings also support the DLE’s utility in capturing a broad experience of racism-related stress. However, unlike earlier studies among college students, we observed weaker associations between the DLE and mental health outcomes of depressive symptoms or perceived stress. Differences in study populations may explain this discrepancy. Previous validation work focused on emerging adult populations, primarily college students aged 18–24 years [16], whereas our sample consisted of women aged 18–35 years who had recently given birth. In addition to being older on average, our participants were navigating the unique physiological, emotional, and social transitions associated with pregnancy and childbirth. While young adults may experience microaggressions within the context of identity development and education and career aspirations [40, 41], pregnant individuals may process these experiences amid complex shifts in not only identity, health, and increased sense of responsibility, but also physiological and psychological responses [42–44]. Such factors could attenuate direct associations with mental health symptoms, partially explaining the weaker convergence observed in our study compared to prior. Additionally, earlier findings emphasized statistical significance over the strength of association [16]. Notably, our findings are consistent with another study validating an alternative measure of microaggressions, the Everyday Discrimination Scale, which also did not find strong correlations with mental health outcomes [45].

Both DLE scales demonstrated strong discriminant validity by showing low correlations with measures of self-esteem, self-kindness and collective hope. These findings suggest that the DLE scales capture constructs that are unique from personality traits or general optimism which could influence how individuals respond to survey questions [33]. To date, this is the first study to examine the discriminant validity of the DLE, adding to the growing evidence that this scale measures a unique and meaningful dimension of experiences of discrimination.

### Public Health Implications

Findings support the reliability and validity of the DLE among a Black postpartum population. Perinatal providers may find the DLE-F useful in understanding their patients experiences of microaggressions across the life course, experiences which have been shown to lead to poor mental and physical health outcomes [18]. Further, researchers examining the health effects of racism and discrimination can consider incorporating the DLE into studies to better capture the lived experiences of Black women. Of note, given the high internal consistency and the unidimensional structure, there may be potential to develop and test a shortened version of the DLE-F.

As it specifically pertains to the DLE-PNC-F, our development and validation of an adapted DLE scale specific to prenatal care provides preliminary evidence for measuring racism-related stress in healthcare contexts. Racial microaggressions encountered during prenatal care likely differ from those in general life, not only in content but also in consequence, because they occur in a setting where trust, communication, and clinical decision-making can directly affect maternal and infant health [18, 21, 22, 29, 30]. Our findings suggest that the DLE-PNC-F has a unidimensional structure with modest fit and strong internal consistency, comparable to the original DLE-F. While further validation is needed, this adapted scale offers a context-sensitive approach that could help researchers examine associations between healthcare discrimination and perinatal outcomes.

### Limitations

The current analysis is subject to a few limitations. First, the generalizability of our findings is limited to Black women during immediate postpartum and results may not extend to other populations or time periods. Second, we did not cross-validate the factor structure or psychometric properties of the scale in an independent sample. As a result, findings regarding the scale’s construct validity, including evidence of divergent validity, should be interpreted cautiously and confirmed in future research using replication. Third, while there are no strict guidelines for cut-offs to use when determining convergent and discriminant validity, we used a potentially generous cut-off of 0.4; a more rigid value may have produced different results [46]. Fourth, a common strategy when testing discriminant validity is to compare scales to level of social desirability [33]. This measure was not available in the current dataset so there is a possibility that the DLE may be influenced by social desirability bias in this sample.

## Conclusions

The present study suggests that the DLE scale, both in its original form (DLE-F) and in a modified version referencing prenatal care (DLE-PNC-F), demonstrate reliability and preliminary validity for measuring experiences of microaggressions among Black postpartum women. The scales consistently demonstrated a single-factor structure and strong internal consistency. Findings indicate that the DLE and DLE-PNC-F may be useful tools for researchers and potentially, with further validation, for perinatal clinicians aiming to better understand and document Black women’s experiences of racial microaggressions and their impact on health and well-being.

## Supplementary Material

ONLINE RESOURCE 1. Scales used for validity assessments

**Supplementary Information** The online version contains supplementary material available at https://doi.org/10.1007/s40615-025-02777-z.

## Figures and Tables

**Table 1:** Participant characteristics for analytical sample with data on either DLE scale (*n* = 514)

Sociodemographic characteristics	*N*	%
Age		
18–20	47	9.1
21–25	152	29.6
26–30	185	36.0
31+	128	24.9
Missing	2	
Marital Status		
Married/living with a partner	220	42.8
Unmarried	294	57.2
Education		
High school diploma or equivalent	429	83.5
No high school degree	70	13.6
Missing	15	
Family Income		
<$10,000	168	32.7
$10,000–$29,999	146	28.4
$30,000–$59,999	123	23.9
$60,000+	71	13.8
Missing	6	1.2
Sought prenatal care		
Yes	479	93.2
No	31	6.0
Missing	4	
DLE Scales	**Mean**	**Standard Deviation**
DLE-F (ranges 20–120)	39.1	20.7
DLE-PNC-F (ranges 17–102)	24.6	14.9

*DLE* Daily Life Experiences, *PNC* prenatal care

**Table 2:** Results from confirmatory factor analysis^a^

Scale	Factors	X^2^	RMSEA	CFI/TLI	SRMR
DLE-F (*n* = 487)	1	889.72	0.13 (0.12–0.14)	0.90/0.88	0.04
DLE-PNC-F (*n* = 478)	1	568.87	0.19 (0.17–0.20)	0.88/0.86	0.03

*DLE* Daily Life Experiences, *PNC* prenatal care, X Chi-square test; *RMSEA* Root Mean Square Error of Approximation, *CFI* Comparative Fit Index, *TLI* Tucker-Lewis Index, *SMSR* standardized root mean square residual

a Model fit was determined to be “good” if: RMSEA 0.7, CFI > 0.90, TLI > 0.90, and SMSR < 0.06

**Table 3:** Standardized factor loadings for one-factor structure models for the DLE scales

	DLE-F	DLE-PNC-F
1. Being ignored, overlooked, or not given service (in a restaurant, store, etc.)	0.838	0.903
2. Being treated rudely or disrespectfully	0.873	0.935
3. Being accused of something or treated suspiciously	0.866	0.907
4. Others reacting to you as if they were afraid or intimidated	0.872	0.942
5. Being observed or followed while in public places	0.831	0.943
6. Being treated as though you were “stupid”	0.877	0.931
7. Your ideas or opinions being minimized, ignored, or devalued	0.875	0.952
8. Overhearing or being told an offensive joke or comment	0.891	0.954
9. Being insulted, called a name, or harassed	0.870	0.946
10. Others expecting your work to be inferior	0.891	---
11. Not being taken seriously	0.915	0.944
12. Being left out of a conversation or activities	0.900	0.958
13. Being treated in an “overly” friendly superficial way	0.876	0.900
14. Being avoided, others moving away from you physically	0.893	0.933
15. Being mistaken for someone who serves others (i.e. secretary, janitor, maid)	0.819	0.943
16. Being stared at by strangers	0.797	0.856
17. Being laughed at, made fun of, or taunted	0.865	0.943
18. Being mistaken for someone else of your same race (who may not look like you at all)	0.857	---
19. Being asked to speak for or represent your entire racial/ethnic group (e.g. “What do __ people think?”)	0.845	0.928
20. Being considered fascinating or exotic by others	0.797	---

*DLE* Daily Life Experiences, *PNC* prenatal care

**Table 4:** Convergent and discriminant validity of the DLE Scales^a^

	DLE-F	DLE-PNC-F
	Rho^b^	Rho^b^
Comparison for Convergent Validity		
Major Experiences of Discrimination	0.53**	0.38**
Vigilance	0.36**	0.30**
Anticipatory Racism Related Stress	0.46**	0.28**
Depressive symptoms (CESD)	0.21**	0.23**
Perceived stress (PSS)	0.24**	0.17**
Comparison for Discriminant Validity		
Self-esteem	0.14**	0.15**
Self-kindness	0.17**	0.15**
Collective hope	0.07	0.06

* *p* < 0.05;

** *p* < 0.01

*DLE* Daily Life Experiences, *PNC* prenatal care, *CESD* Centers for Epidemiologic Studies Depression, *PSS* Cohen’s Perceived Stress Scale

a See Online Resource 1. for details on the scales

b Absolute value of rho presented and compared
