# Supplementary material for: Measurement of Racial Microaggressions During Pregnancy and While Receiving Prenatal Care: a Validation Study of the Daily Life Experiences Scale Among Black Postpartum Women
Source: J Racial Ethn Health Disparities. Author manuscript; Available in PMC 2026 Jan 29. (PMC12854132; doi:10.1007/s40615-025-02777-z)
Supplement: ONLINE RESOURCE 1. Scales used for validity assessments [file NIHMS2140602-supplement-ONLINE_RESOURCE_1__Scales_used_for_validity_assessments.pdf]

ONLINE RESOURCE 1. Scales used for validity assessments

Assessment	Scale Name	Scale Items
Convergent Validity	Williams' Major Experiences of Discrimination	1. At any point in your life, have you ever been unfairly fired?
		2. For unfair reasons, have you ever not been hired for a job?
		3. Have you ever been unfairly denied a promotion?
		4. Have you ever been unfairly stopped, searched, questioned, physically threatened, or abused by the police?
		5. Have you ever been unfairly discouraged by a teacher or advisor from continuing your education?
		6. Have you ever been unfairly prevented from moving into a neighborhood because the landlord or a realtor refused to sell or rent you a house or apartment?
		7. Have you ever moved into a neighborhood where neighbors made life difficult for you or your family?
		8. Have you ever been unfairly denied a bank loan, credit card, or line of credit?
		9. Have you ever received service from someone such as a retail service worker, restaurant employee, or car repair person that was worse than what other people get?
	Anticipatory Racism-Related Stress	1. When I am around White people, I expect them to say or do something racist
		2. I believe that most Black people will experience some form of racism in the future
		3. I know if I go where there are mostly White people, there is a good chance I will experience racism
		4. I believe there is a good chance that I will experience racism in the future
	Vigilance	1. Try to prepare for possible insults from other people before leaving home
		2. Feel that you always have to be very careful about your appearance to get good service or avoid being harassed
		3. Carefully watch what you say and how you say it
		4. Try to avoid certain social situations and places
	Centers for Epidemiologic Studies Depression (CESD) Scale	1. I was bothered by things that don't usually bother me
		2. I had trouble keeping my mind on what I was doing
		3. I felt depressed
		4. I felt that everything I did was an effort
		5. I felt hopeful about the future
		6. I felt fearful
		7. My sleep was restless
		8. I was happy
		9. I felt lonely
		10. I could not "get going"
	Cohen's Perceived Stress Scale (PSS)	1. How often have you been upset because of something that happened unexpectedly?
		2. How often have you felt that you were unable to control the important things in your life?
		3. How often have you felt nervous and "stressed"?
		4.How often have you felt confident about your ability to handle your personal problems?
		5. How often have you felt that things were going your way?
		6. How often have you found that you could not cope with all the things that you had to do?
		7. How often have you been able to control irritations in your life?

		8. How often have you felt that you were on top of things?
		9. How often have you been angered because of things that were outside of your control?
		10. How often have you felt difficulties were piling up so high that you could not overcome them?
Discriminant Validity	Rosenberg Self-Esteem Scale	1. I take a positive attitude toward myself
		2. On the whole, I am satisfied with myself
		3. I certainly feel useless at times
		4. At times I think I am no good at all
	Unconditional Self-kindness Scale	1. How much are you patient and tolerant with yourself when you are criticized or rejected by another person
		2. How much are you loving and kind to yourself when you become aware of your personal flaws and imperfections?
		3. How much are you patient and tolerant with yourself when you fail or make a mistake?
		4. How much are you loving and kind to yourself when you are criticized or rejected by another person?
		5. How much are you patient and tolerant with yourself when you become aware of your personal flaws and imperfections?
		6. How much are you loving and kind to yourself when you fail or make a mistake?
	Collective Hope (difference between responses)	1. Pretend that the steps on the ladder below are 10 possible steps towards racial equality in the United States. The 10th step at the top stands for total equality for Black Americans (equal employment opportunities, housing, education, etc.) while the 1st step at the bottom stands for total inequality for Black Americans. Please tell us the step # that best describes where Black people living in the US are today.
		2. Please tell us the step # that best describes where Black people living in the US will be in 5-10 years from now.
